# Comparison of High-Pressure Behavior of Physicochemical Properties of the Di- and Triacylglycerols Established by Ultrasonic Methods

**DOI:** 10.1007/s11746-017-3030-y

**Published:** 2017-08-30

**Authors:** A. Balcerzak

**Affiliations:** Section of Acoustoelectronics, Institute of Fundamental Technological Research, Polish Academy of Sciences, ul. Pawińskiego 5B, 02-106 Warsaw, Poland

**Keywords:** Acylglycerols, Physicochemical parameters, Ultrasonic measurements

## Abstract

Two samples of triacylglycerols i.e., olive oil and triolein, and one sample of diacylglycerol were investigated. In the course of compression, the density of the samples was determined by measurements of the change of piston position in a pressure chamber and volume correction due to chamber expansion under pressure. The speed of sound was evaluated from the time of flight of an ultrasonic impulse between emitting and receiving transducers placed in the high pressure chamber. The adiabatic compressibility, the intermolecular free length, the molar volume, the van der Waals’ constant *b* and the surface tension were evaluated from the density, the speed of sound and the average molecular mass. All tested liquids undergo a high-pressure phase transition. Discontinuities in the measured isotherms of the physicochemical parameters of the investigated oils indicate the presence of high-pressure phase transitions. Moreover the time dependent change of pressure at constant volume during the phase transition was measured. The fundamental difference in the molecular structure of these acylglycerols influences their behavior significantly under high pressure.

## Introduction

Acylglycerols are esters of glycerol and fatty acids. Depending on the number of the esterified hydroxyl groups in glycerol, the molecules of acylglycerols can contain three, two or one fatty acid moieties. This class of chemical compounds has a fundamental importance as foodstuffs and as components for production of surfactants, emulsifiers and biofuels. Their signification for the fuel industry is due to the possibility of their conversion to methyl- or ethyl esters and their use as fuel for diesel engines.

High-pressure physicochemical properties of acylglycerols play an important role in food, chemical and fuel technological processing since the extensive application of high pressure processes for their conversion, conservation and preservation [1–4]. Lack of data for these parameters is a great problem in the design and optimization of technological processes. Physicochemical properties of acylglycerols and their changes caused by high hydrostatic pressure are important for the study of molecular structures and interactions of acylglycerols. Among others this kind of study becomes significant for acylglycerol metabolism and nutritional values due to increasing production of high-pressure preserved foodstuffs.

Sound speed is linked to many physicochemical parameters of liquids. Evaluation of physicochemical parameters of liquids under high pressure is extremely difficult. On the other hand sound speed can be relatively easily measured at high pressure. In this way, from high-pressure measurements of the sound speed and density, the pressure dependent physicochemical parameters of liquids can be evaluated.

The aim of this work was to measure density and sound speed of acylglycerols under high-pressure, and to evaluate the resulting physicochemical parameters such as: adiabatic compressibility, intermolecular free length, molar volume, van der Waals’ constant *b* and surface tension, and consequently to compare the high-pressure behavior of triacylglycerols (TAG) and diacylglycerol (DAG). Knowledge of the behavior of the physicochemical parameters during high pressure compression and possible occurrence of phase transitions is valuable from scientific (molecular structures and their changes in the course of pressure increasing) and technological (designing of chemical, fuel and food installations and their proper operation) points of view.

By using ultrasonic methods we revealed the occurrence of a high-pressure phase transition in the investigated liquids. Discontinuities in the measured isotherms of density and sound speed versus pressure in all investigated liquids indicated the presence of liquid to solid-like transformations.

## Materials

### TAG Samples

Triacylglycerols are esters of the trihydric alcohol glycerol in which all three hydroxyl groups are esterified with fatty acids. Two kinds of TAG were used for measurements:

- Triolein (Sigma-Aldrich, practical grade >65%).
- Olive oil.

This olive oil was produced in Spain with a high content of triacylglycerols (97%) of oleic acid mainly (76.8% of all acids)—Table 1 [5]. The remainder (3%) were DAG, MAG, free acids and other compounds. In investigated olive oil, the dominant component was oleic acid (C18:1 *cis*)—Table 1 [5]. The second, third, fourth and fifth main components were palmitic (C16:0) and linoleic (C18:2 *cis*–*cis*), stearic (C18:0) and palmitoleic (C16:1) acids, respectively. Other fatty acids were present in small amounts (<1%).

**Table 1:** Chemical composition of investigated olive oil

Fatty acid	C14:0	C16:0	C16:1	C17:0	C17:1	C18:0	C18:1c	C18:2ct/tc
% in olive oil	<0.1	11.02	1.01	0.1	0.1	3.4	76.8	<0.1
Fatty acid	C18:2cc	C18:3ccc	C20:0	C20:1	C20:2	C22:0	C24:0	C24:1
% in olive oil	5.9	0.7	0.4	0.3	<0.1	0.1	<0.1	<0.1

The analysis of oils were made by the Institute of Agricultural and Food Biotechnology, Warsaw, Poland. The composition of the tested oil was analyzed by means of a gas chromatography method using a Hewlett-Packard HP 6890 device with a Flame Ionization Detector and a high-polar column BPX70. This analysis was made following the AOCS Cd 11b-91 method and was performed according to the ISO 5508 and ISO 5509 norms.

### DAG Sample

Diacylglycerols are esters of the trihydric alcohol glycerol in which two of the hydroxyl groups are esterified with fatty acids.

DAG oil used for testing, obtained from the Institute of Agricultural and Food Biotechnology, Warsaw, Poland, was composed of 82% diacylglycerols (DAG) and 17.9% of triacylglycerols (TAG), with a small content (0.1%) of monoacylglycerols and other compounds. The prevailing component was oleic acid (C18:1 *cis*)—Table 2 [6]. The second, third, fourth, fifth and sixth main components were linoleic (C18:2 *cis*–*cis*), linolenic (C18:3 *cis*–*cis*–*cis*), palmitic (C16:0), stearic (C18:0) and arachidic (C20:0) acids, respectively. Other fatty acids were present in small amounts (≤1.2%).

**Table 2:** Chemical composition of tested DAG oil

Fatty acid	C14:0	C16:0	C16:1	C17:0	C17:1	C18:0	C18:1c	C18:2cc
% in DAG	0.1	4.5	0.5	0.1	0.1	2.5	59.1	19.6
Fatty acid	C18:3ccc	C20:0	C20:1	C20:0	C22:1	C24:0	C24:1	
% in DAG	8.9	1.9	1.0	0.1	1.2	0.3	0.1	

The analysis was conducted analogically as for the TAG sample.

## Methods

A detailed description of the high-pressure measuring setup is presented in [7–11]. Pressure in the testing chamber was produced by a manually driven hydraulic press. Pressure and temperature sensors were placed inside the pressure chamber within the investigated oil sample. The volume of chamber equals 22 cm^3^. The change of electrical resistance of calibrated manganin sensor was used to measure the pressure inside the chamber. Volume changes of the sample under pressure were calculated from the piston displacement registered by a digital caliper. Corrections related to the chamber expansion were evaluated from the Lame equations. These two facts allowed the calculation of the density of the sample under pressure since the mass of the sample was not changed during the measurements. The sample density under atmospheric pressure was determined by means of a U-shaped tube densitometer (MG-2, Unilab, Poland). The temperature was kept at 20 °C ± 0.05 °C by a circulation of thermostatic liquid in the jacket of the chamber supplied by a precision thermostat (Julabo Labortechnik, Seelbach, Germany).

The pressure inside the chamber was measured by a 100 Ω manganin wire sensor [12]. The resistance of the manganin wire sensor was measured using an NI 4065 multimeter computer card (National Instruments Corp., Austin, TX, USA). The pressure was increased in 10 MPa/0.5 min steps and with a time interval sufficient to establish the thermodynamic equilibrium of the liquid sample—1 min. All measurement data were collected every 10 s. The measurements of every sample was repeated three times.

**Fig. 1:**
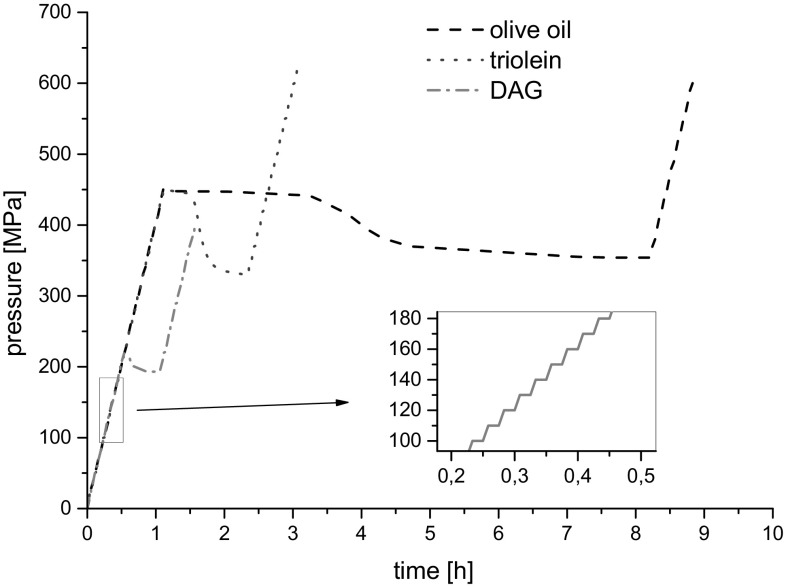
Plots of the time dependences of pressure at 20 °C

**Fig. 2:**
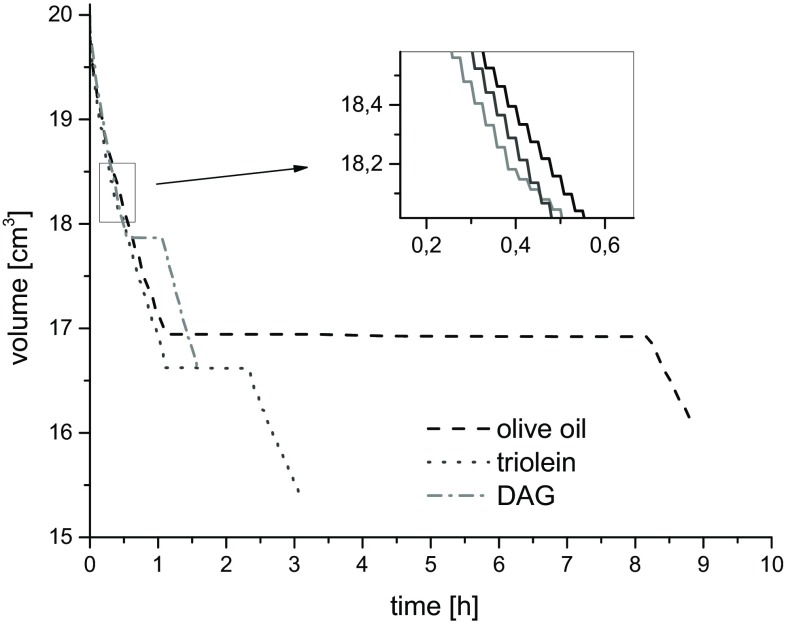
Plots of the time dependences of sample volume at 20 °C

The time dependence of pressure and volume changes are shown in Figs. 1 and 2. In the low-pressure phase and high-pressure phase regions, the volume of the sample decreased stepwise following the pressure increase. During the phase transition pressure decreases spontaneously at almost constant volume.

Before measurements, the setup was calibrated by using distilled water as the reference liquid. Subsequently exact values of the distance between transducers were calculated using known values of the water speed of sound as a function of pressure.

The speed of sound was determined from the time of flight of ultrasonic waves between two transducers. The sending and receiving transducers were made from two 5 MHz LiNbO3 plates (Y36 cut, Boston Piezo-Optic Inc., Bellingham, MA, USA). The sending transducer was driven by a signal from the TB-1000 pulser-receiver computer card (Matec Instrument Companies Inc., Northborough, MA, USA). The electric signal was changed in the ultrasonic one by the sending transducer. Then the ultrasonic signal passed through the sample of the investigated liquid and was changed into an electrical signal by the receiving transducer. This signal was processed by the PDA-1000 digitizer computer card (Signatec, USA). To increase the signal-to-noise ratio, each single measurement was repeated 1024 times and averaged. The cross-correlation method [13] was applied to evaluate the time of flight for the signal. The experimental setup was calibrated by measurements made for distilled water.

The speed of sound, *c*, was calculated from Eq. 1:

1 $$c = \frac{l}{t} \;\left( {{\text{m}}/{\text{s}}} \right)$$

where *l* is the distance between transducers and *t* is the time of flight for the signal.

The time of flight of ultrasonic waves, *t*, was measured with picosecond resolution. However, due to extra systematic errors, such as diffraction contribution, the resulting relative standard uncertainty of time measurements was estimated as ±0.1%. Similarly, the relative standard uncertainty of the distance between transducers, *l*, (resulting from calibration measurements in distilled water) was estimated as ±0.1%. According to the ISO guidelines the expanded relative uncertainty of the ultrasonic velocity is ±0.3% at a 95% confidence level.

The main source of error in determining the volume of the measured sample was the piston displacement measurement error. The piston displacement was measured using a digital caliper with an uncertainty of ±0.01 mm. This gave volume measurement relative standard uncertainty ±0.03%. The expanded relative uncertainty of the density was ±0.05%.

The pressure measurements uncertainty was equal ±0.1 MPa.

## Results and Discussion

### Density

**Fig. 3:**
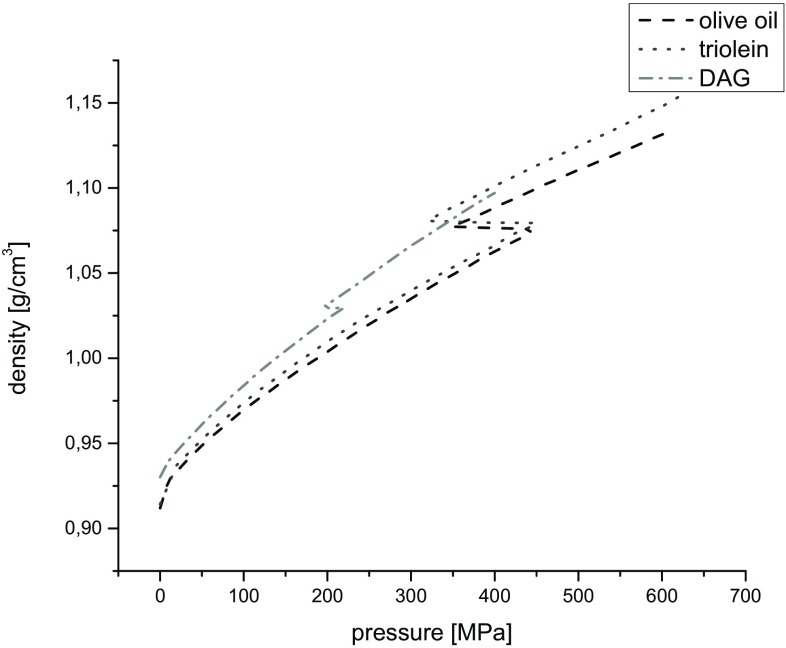
Plots of densities as a function of pressure at 20 °C

Results of the measurements of density, *ρ*, as a function of pressure, are presented in Fig. 3.

The density of all investigated liquids increases with pressure and was the highest in the high-pressure region. The phase transition of DAG occurred at lower pressure (~220 MPa) compared to olive oil (~448 MPa) and triolein (~450 MPa). The density of DAG was higher than that of triolein and olive oil in liquid state but was lower than that of triolein in the solid-like state. That fact is a consequence of the molecular structure of the DAG which was smaller than TAG. However, the solid structure of the olive oil and triolein are more compact than that of the DAG. Density was almost constant during the phase transformation, because the piston shift indicated by the caliper did not change. Thus, this transformation is an isochoric one under the experimental conditions.

### Speed of Sound

**Fig. 4:**
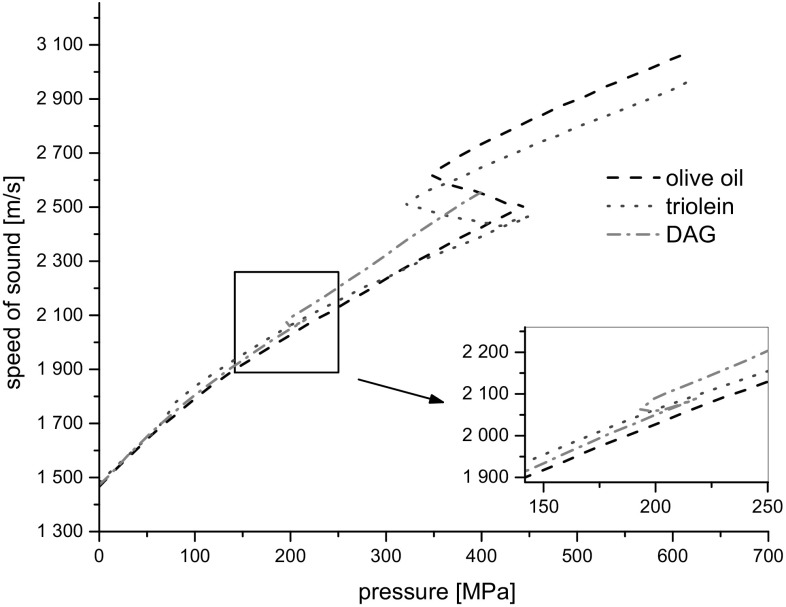
Plots of speed of sound as a function of pressure for investigated liquids at 20 °C

Results of the measurements of the speed of sound, *c*, as a function of pressure, are presented in Fig. 4.

In the case of speed of sound, all tested liquids exhibited quantitative similarity up to phase transformation while they were compressed. In phase transformation the speed of sound for triolein initially decreased following the low-pressure phase compression curve (from 450 to 420 MPa). Subsequently the speed of sound increased and the pressure decreased spontaneously. The speed of sound for the olive oil increased in the entire pressure range in the phase transition region. The behavior of DAG was similar to that for TAG up to the point of beginning of the DAG phase transition. Subsequently, in the high-pressure phase region, the plots of the speed of sound for DAG and TAG diverged. In the phase transition region, the change in the sound speed in DAG sample was significantly smaller than that in TAG samples.

The remarkably smaller speed of sound for the solid state of DAG can be argued as being due to its less compact structure compared to triolein and olive oil. This observation is in agreement with the density of solid DAG which is smaller than the density of triolein. Interestingly, the speed of sound of solid-like olive oil was highest among others. This may suggest that the solid-like structure of olive oil is the most compact and has more solid content. This can be attributed to its highest content of saturated fatty acids (~14.42%) that consisted of palmitic and stearic acids, while DAG oil consisted of ~7% similar saturated fatty acids.

### Adiabatic Compressibility

The adiabatic compressibility of a liquid, *β*_S_, is calculated as:

2 $$\beta_{\text{S}} = \frac{1}{{c^{2} \rho }}\; \left( {{\text{kg}}^{ - 1} {\text{s}}^{ 2} {\text{m}}} \right).$$

**Fig. 5:**
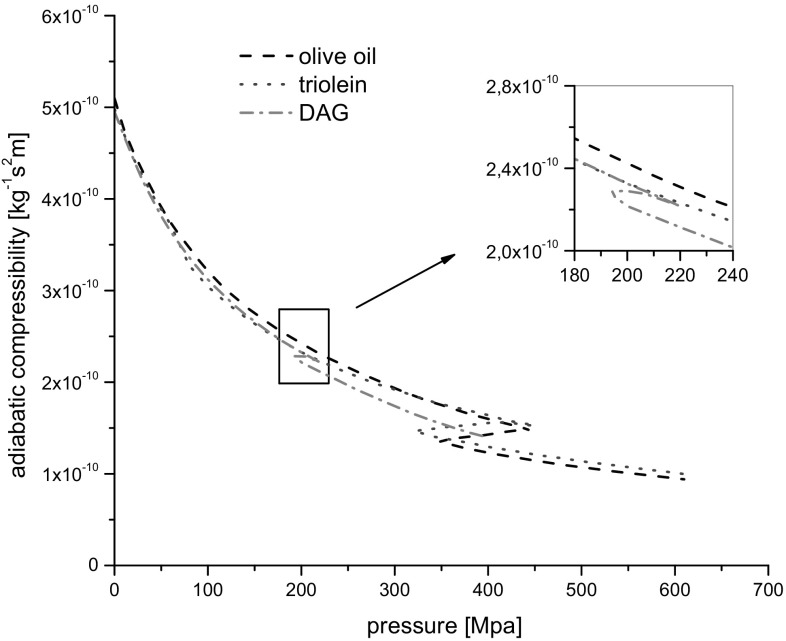
Adiabatic compressibility as a function of pressure at 20 °C

The obtained results are presented in Fig. 5. The relative uncertainty of the adiabatic compressibility was ±0.7%.

From comparison of Figs. 4 and 5 one can conclude that the influence of density changes did not change significantly the behavior of adiabatic compressibility when pressure increased.

The high adiabatic compressibility values of the solid DAG can further support the facts that the solid-like structure of DAG is less compact and possess a larger free volume then the TAG. This can also be true because the phase transition of DAG occurred at low pressure level compared to olive oil and triolein. The adiabatic compressibility of olive oil was lower than that of triolein what may suggest that the solid-like structure of olive oil has less free space (volume without molecules) (Fig. 9).

### Intermolecular Free Length

The intermolecular free length, *L*_f_, was calculated from the formula:

3 $$L_{\text{f}} = \frac{K}{{c\rho^{{{1 \mathord{\left/ {\vphantom {1 2}} \right. \kern-0pt} 2}}} }} = K\sqrt {\beta_{\text{S}} } \; \left( {\text{m}} \right)$$

where *K* is the Jacobson constant determined by the dependence *K* = (93.875 + 0.375 *T*) × 10^−8^ [(m kg)^1/2^/s]. *T* denotes absolute temperature in Kelvin. The relative uncertainty of the intermolecular free length was ±0.3%.

**Fig. 6:**
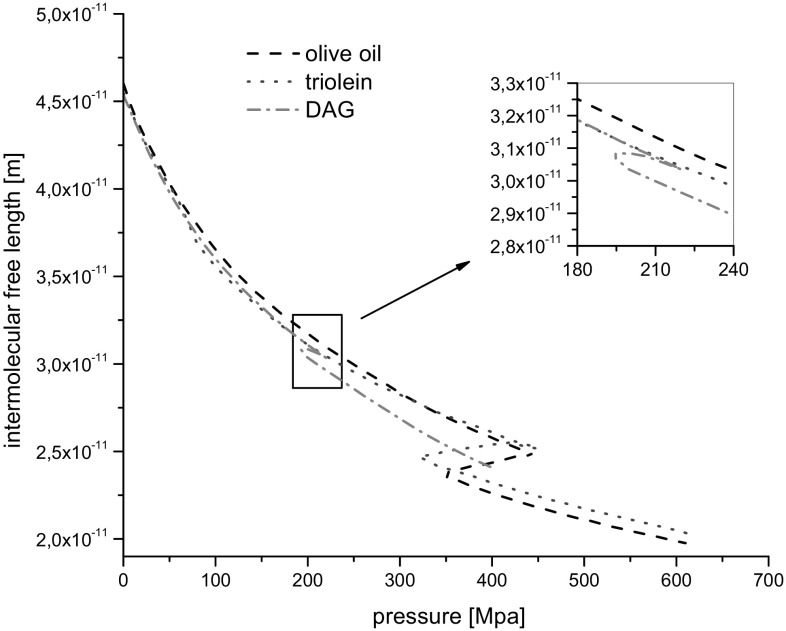
Intermolecular free length *vs* pressure at 20 °C

The dependence of *L*_f_ on pressure is shown in Fig. 6.

The intermolecular free length, *L*_f_, decreased monotonously when the pressure increased, except in the region of phase transformation. For the low range of pressure before the phase transformation the plots of *L*_f_ almost overlapped for all investigated liquids. Distances between molecules became shorter with increasing pressure what suggests that the solid-like structures were still compressible but at lower values. The intermolecular free length of olive oil was lower compared to triolein which supports the fact that free space in the solid-like structure of olive oil was lower. This can be attributed to a higher percentage of unsaturated fatty acid in triolein compared to that of olive oil what contributes to more steric hindrance. In the case of DAG the different behavior can be noticed in the phase transformation region. Here the intermolecular free length slightly increased and pressure decreased. Perhaps these different behaviors of both TAG compared to that of DAG and the greater pressure change during phase transition for TAG than in the case of DAG were caused by smaller steric hindrances in DAG structure than in TAG one. This could be due to the fact that the molecules of TAG contained one more moiety of the long chain fatty acid than DAG molecules.

### Molar Volume

**Fig. 7:**
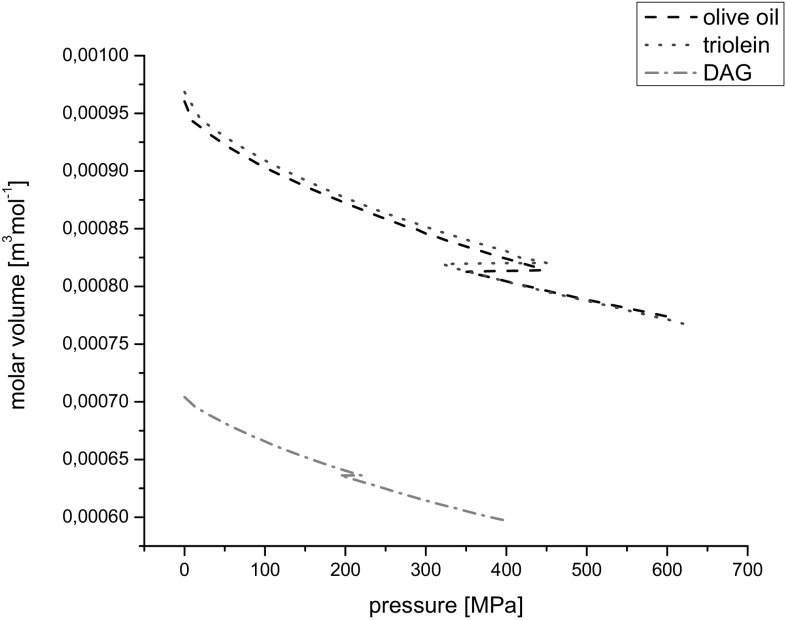
Molar volume *vs* pressure at 20 °C

The molar volume, *V*_M_, was calculated according to the formula:

4 $$V_{\text{M}} = \frac{M}{\rho }\;\left( {{\text{m}}^{ 3} /{\text{mol}}} \right)$$

where *M* is the molar mass (see Fig. 7). The relative uncertainty of the molar volume was ±0.05%.

Mean molar masses of investigated liquids i.e. olive oil, triolein and DAG were calculated from the mass ratios and molecular masses of the components and were equal to 0.8756, 0.8855 and 0.6550 kg mol^−1^, respectively.

### Van der Waals’ Constant

**Fig. 8:**
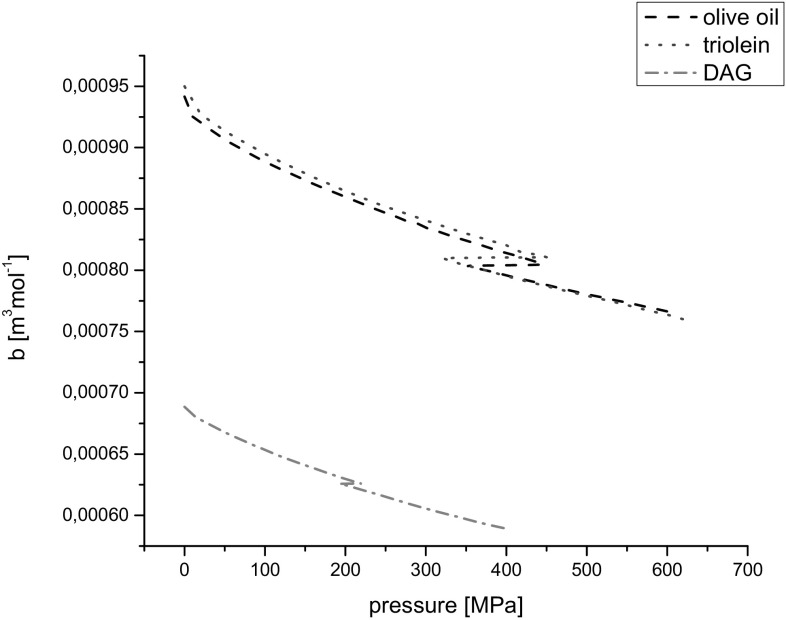
Dependence of van der Waal’s constant *b* on pressure at 20 °C

The van der Waals’ constant, *b*, which takes account of the molecule dimensions and is the volume of one mol of molecules only, is determined from:

5 $$b = \frac{M}{\rho }\left\{ {1 - \left( {\frac{RT}{{Mc^{2} }}} \right)\left[ {\left( {1 + \frac{{Mc^{2} }}{3RT}} \right)^{{{\raise0.7ex\hbox{$1$} \!\mathord{\left/ {\vphantom {1 2}}\right.\kern-0pt} \!\lower0.7ex\hbox{$2$}}}} - 1} \right]} \right\}\; \left( {{\text{m}}^{ 3} {\text{mol}}^{ - 1} } \right)$$

where *R* = 8.314 J K^−1^ mol^−1^ is the universal gas constant. The changes of values of parameter *b* on pressure are presented in Fig. 8. The relative uncertainty of the van der Waals’ constant was ±1%.

For triacylglycerols i.e. olive oil and triolein, values of the parameter b and their high-pressure behavior were similar. This fact could be understood in terms of both oils structures comprising mainly unsaturated triacylglycerols. The molecules of DAG are smaller because they contain two moieties of fatty acids. Therefore, for the DAG oil, the values of the parameter b were lower than those for olive oil and triolein.

It is worth comparing the product of subtraction of molar volume *V*_M_ and van der Waals’ constant *b* for the tested liquids:

6 $$V_{\text{A}} = V_{\text{M}} - b\;\left( {{\text{m}}^{ 3} {\text{mol}}^{ - 1} } \right).$$

**Fig. 9:**
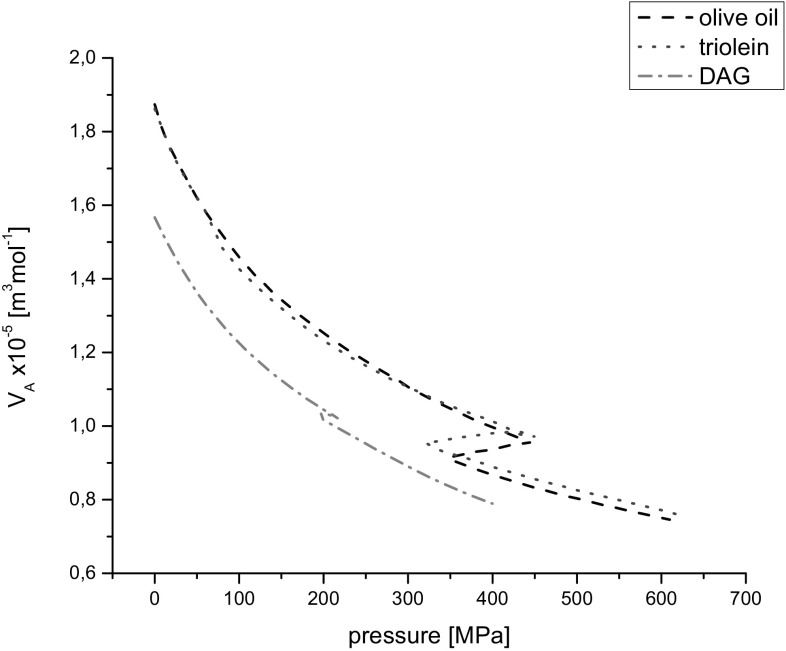
Parameter *V*_A_ *vs* pressure at 20 °C

Values of *V*_A_ are presented in Fig. 9. According to formula (6), *V*_A_ can be considered as volume without molecules for one mole of a substance. Analyzing the plots in Fig. 9 one can see that for olive oil and triolein the plots of *V*_A_ against pressure are similar in the regions of the low-pressure phase and high-pressure phase. During phase transition the volume without molecules, *V*_A_, decreased and values of *V*_A_ were lower for olive oil than for triolein. This can be related to the composition of both oils. For DAG the values of *V*_A_ were distinctly lower than those for triacylglycerols. In the region of phase transition the volume without the volume of molecules themselves, *V*_A_, for DAG oil slightly increased, because the molecules of DAG components according to their simpler structures were more tractable on pressure constraint than olive oil and triolein.

### Surface Tension

Surface tension was calculated with the Auerbach formula [14]:

7 $$\sigma = 6.33 \times 10^{ - 10} \rho c^{{{\raise0.7ex\hbox{$3$} \!\mathord{\left/ {\vphantom {3 2}}\right.\kern-0pt} \!\lower0.7ex\hbox{$2$}}}} \; \left( {{\text{N}}/{\text{m}}} \right).$$

**Fig. 10:**
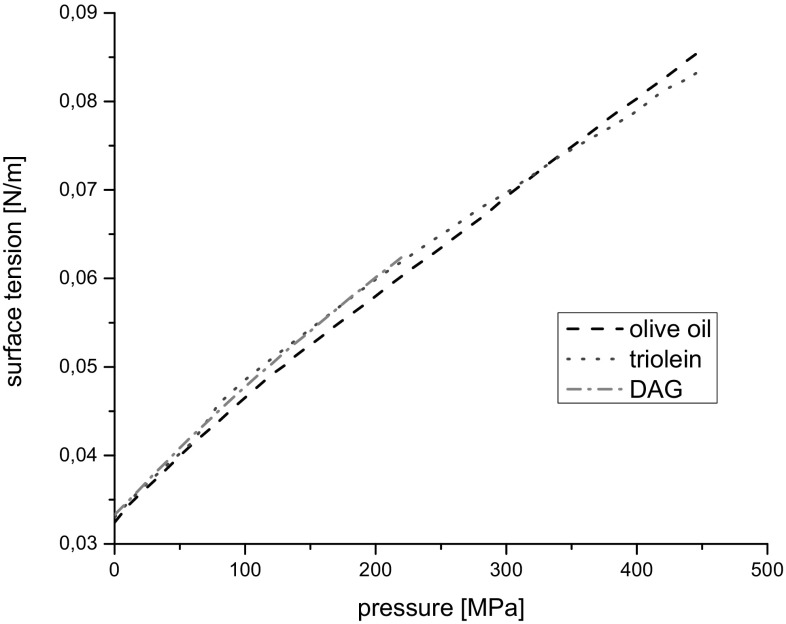
Surface tension *vs* pressure at 20 °C

The obtained results for the investigated liquids are shown in Fig. 10. The relative uncertainty of the surface tension was ±0.5%.

Despite the difference in molecular structure of the investigated liquids the values of the surface tension, *σ*, in the course of pressure increasing, were similar for both TAG and DAG oil.

### Dependence of pressure on time during high-pressure phase transitions

**Fig. 11:**
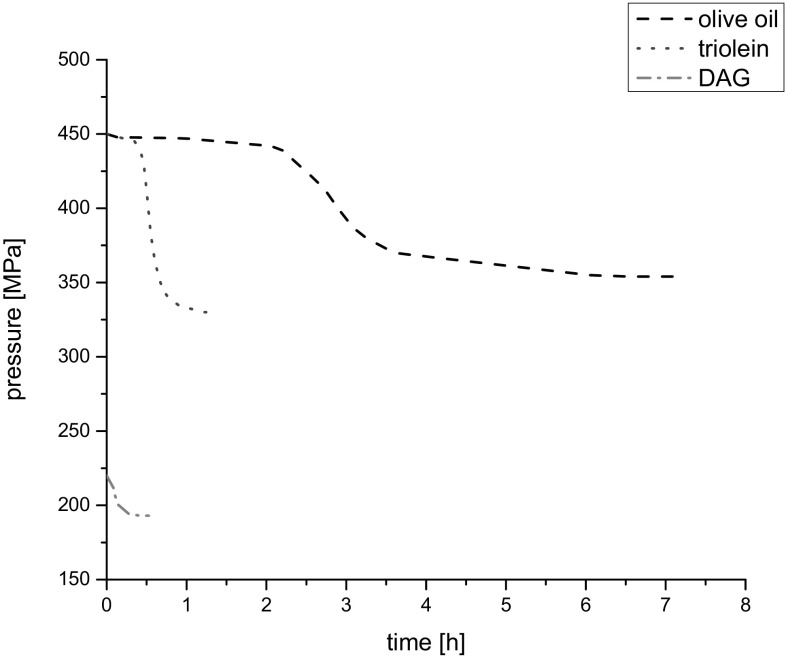
Dependences of pressure on time in the course of phase transition at 20 °C

The dependences of pressure on time during phase transition for all investigated liquids are shown in Fig. 11.

Great differences in the behavior of tested liquids in the phase transition region can be easily noted. For triolein the decrease in pressure was significantly sharper than for olive oil. In olive oil the time before and after the curve inflection was longer than for triolein. Thus the molecules of the components of olive oil need more time to start the phase transition and achieve the equilibrium state than that of triolein ones. For DAG the phase transition was faster and with low change of pressure. The molecular rearrangement of structure containing two fatty acid moieties (DAG) needs shorter time than these with three fatty acid moieties (olive oil and triolein) due to greater complication of molecular structure and steric obstacles.

## Conclusions

The origin of this study was motivated by the lack of sufficient information on physicochemical parameters of liquids and changes in their molecular structures under high-pressure conditions. High-pressure physicochemical properties of acylglycerols play an important role in food, chemical and fuel industries since the extensive application of high pressure processes for their conversion, conservation and preservation.

From the ultrasonic measurements in a wide range of hydrostatic pressure the main parameters i.e., density and speed of sound for considered samples of olive oil, triolein and DAG oil were developed experimentally. The following physicochemical parameters of investigated oils were evaluated: adiabatic compressibility, intermolecular free length, van der Waals' constant *b* and product of subtraction of molar volume *V*_M_, van der Waals' constant *b*, surface tension and dependence of pressure on time during high-pressure phase transitions. The analysis of the results shows great differences in the high-pressure behavior of the physicochemical parameters for TAG and DAG due to their different molecular structure i.e. larger, stiffer and bigger steric hindrances for TAG than that for DAG oil. Small differences of physicochemical parameters between two tested TAG i.e., olive oil and triolein, were observed. This phenomenon can be caused by different content of olive oil and the main triolein component i.e. oleic acid (C18:1 *cis*) 76.8% and >65%, respectively.
